# A Flexible, Wearable, and Wireless Biosensor Patch with Internet of Medical Things Applications

**DOI:** 10.3390/bios12030139

**Published:** 2022-02-22

**Authors:** Duc Tri Phan, Cong Hoan Nguyen, Thuy Dung Pham Nguyen, Le Hai Tran, Sumin Park, Jaeyeop Choi, Byeong-il Lee, Junghwan Oh

**Affiliations:** 1 Industry 4.0 Convergence Bionics Engineering, Pukyong National University, Busan 48513, Korea; 202056824@pukyong.ac.kr (D.T.P.); hoannc@pukyong.ac.kr (C.H.N.); pntdung95@pukyong.ac.kr (T.D.P.N.); tranlehai999@pukyong.ac.kr (L.H.T.); tnlas030980@pukyong.ac.kr (S.P.); eve1502@pukyong.ac.kr (J.C.); 2 BK21 FOUR ‘New-Senior’ Oriented Smart Health Care Education, Pukyong National University, Busan 48513, Korea; 3 Department of Smart Healthcare, Pukyong National University, Busan 48513, Korea; 4 Biomedical Engineering, Pukyong National University, Busan 48513, Korea; 5 Ohlabs Corporation, Busan 48513, Korea

**Keywords:** biosensors, physiological signals, Internet of Medical Things (IoMT), artificial neural network (ANN)

## Abstract

Monitoring the vital signs and physiological responses of the human body in daily activities is particularly useful for the early diagnosis and prevention of cardiovascular diseases. Here, we proposed a wireless and flexible biosensor patch for continuous and longitudinal monitoring of different physiological signals, including body temperature, blood pressure (BP), and electrocardiography. Moreover, these modalities for tracking body movement and GPS locations for emergency rescue have been included in biosensor devices. We optimized the flexible patch design with high mechanical stretchability and compatibility that can provide reliable and long-term attachment to the curved skin surface. Regarding smart healthcare applications, this research presents an Internet of Things-connected healthcare platform consisting of a smartphone application, website service, database server, and mobile gateway. The IoT platform has the potential to reduce the demand for medical resources and enhance the quality of healthcare services. To further address the advances in non-invasive continuous BP monitoring, an optimized deep learning architecture with one-channel electrocardiogram signals is introduced. The performance of the BP estimation model was verified using an independent dataset; this experimental result satisfied the Association for the Advancement of Medical Instrumentation, and the British Hypertension Society standards for BP monitoring devices. The experimental results demonstrated the practical application of the wireless and flexible biosensor patch for continuous physiological signal monitoring with Internet of Medical Things-connected healthcare applications.

## 1. Introduction

The importance of regular physiological metric monitoring and smart data analytics in the early detection and prevention of cardiovascular diseases has been reported [1,2]. Owing to the advantages of printed electronics, flexible devices and wireless body sensor networks have been developed to continuously collect various types of physiological data such as heart rate variability, body temperature, systolic blood pressure (SBP), and diastolic blood pressure (DBP) [3]. The development and advancement of wearable biosensor devices is valuable for improving the self-monitoring compliance of patients and enhancing the quality of the healthcare system [4,5,6,7]. However, monitoring different physiological signals requires multiple biosensors and devices, which increases the cost of equipment and causes inconvenience and discomfort to users [3,8]. Hence, it is important to develop a single device with multiple sensor combinations for continuous physiological signal monitoring [9,10].

With the rise in information and communication technology, the concept of an Internet of Medical Things (IoMT) has attracted growing attention over recent years [11,12]. Advances in IoMT and precision medicine have transformed the healthcare system, particularly in terms of regular and frequent physiological monitoring and cardiac risk assessment [12]. The concept and application of a health Internet of Things and wearable electronics are expected to address limitations of the traditional healthcare system, such as the shortage of medical staff and high medical cost burdens [13]. The IoMT is expected to become a technology that can improve people’s health conditions, potentially increasing the human lifespan and preventing chronic diseases [14].

Regular blood pressure (BP) monitoring is important for patient diagnosis and management [15]. However, conventional ambulatory BP techniques, such as oscillometric measurement, require a cuff to be placed and repeatedly inflated around the upper arm [14,15]. Although oscillometric measurement is the gold standard to extract the continuous features and the for BP monitoring, it causes discomfort to users and does not allow continuous BP monitoring [16]. Motivated by this, several cuffless BP measurement methods for continuous and non-invasive BP monitoring based on the relationship between pulse arrival time and blood pressure have been proposed [17,18,19]. The BP estimation model has been developed morphological characteristics of the input signals. A common technique for calculating pulse arrival time is based on electrocardiogram (ECG) and photoplethysmography (PPG) signals; pulse transit time–BP models are used to estimate BP [20,21]. Furthermore, the emergence of artificial intelligence techniques has great potential for solving complicated systems with high-level features of inputs [22,23]. Machine learning algorithms (support vector machine, random forest) and deep learning techniques have been investigated and have shown superior performance in BP estimation in several studies [24,25,26]. Yung et al. introduced a BP estimation method using a bidirectional layer of long short-term memory (LSTM). They extracted features from ECG and PPG signals from the dataset of Physionet’s Multiparameter Intelligent Monitoring in Intensive Care II (MIMIC-II) to predict systolic blood pressure (SBP) and diastolic blood pressure (DBP). The obtained performance for BP estimation passes the AAMI (Association for the Advanced of Medical Instrument protocols) standard [27]. Monika et al. developed a BP estimation model using only ECG signals and different machine learning models (KNN, SVM, Naïve Bayes, etc.) and a regression module. They predicted SBP and DBP with a mean absolute error (MAE) of 7.72 mmHg for SBP, 9.45 mmHg for DBP, and 8.13 mmHg for MAP [28]. Peng et al. proposed a novel deep recurrent neural network (RNN) for long-term BP prediction. The RNN model was tested and achieved a root mean square error (RMSE) of 3.90 and 2.66 mmHg for SBP and DBP, respectively [29]. However, most pulse transit time–BP estimation models require two channels of physiological signals for calculation, and BP estimation models based on single-channel PPG signals exhibit inferior performance due to motion artifacts (MA) and noise effects [30,31]. Compared to PPG signals, ECG signals are more reliable and display a closer association with BP [32,33]. Therefore, in the present research, a BP estimation model based on artificial neural networks and one-channel ECG signals for unobtrusive and continuous BP monitoring is proposed.

Here, we present a biosensor patch for remote healthcare monitoring. The main objective of this paper is to propose a wearable, wireless, and integrated biosensor device with an IoT-connected healthcare platform for continuous and simultaneous vital signs monitoring. Overall, the main contributions of this paper include: (1) The design and development of a multimodal wearable patch with high wearing comfort, stability for long-term attachment, and wireless connection for health self-monitoring. (2) The introduction of an IoMT platform with a cloud server, smartphone application, and remote monitoring website. (3) Demonstration of the feasibility of the multiple biosensor integration for monitoring different vital signs, which will be necessary for the understanding of the body’s response and self-monitoring compliance. (4) The proposal of a BP estimation model based on a one-dimensional convolutional neural network (1D-CNN) and single-channel ECG signals with superior performance.

## 2. Materials and Methods

### 2.1. A Wireless, Flexible Biosensor Patch with a Healthcare IoT (H-IoT) Application

**Figure 1:**
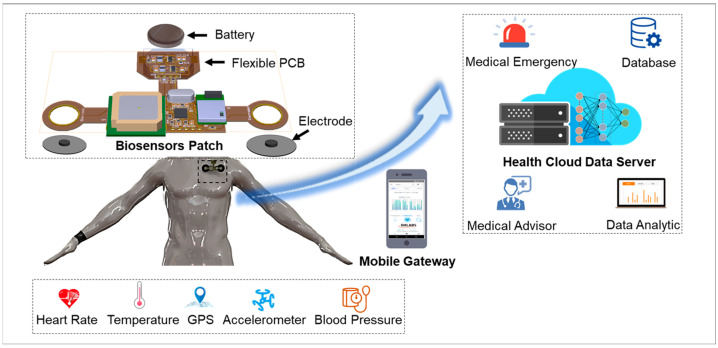
Overview of wearable biosensor patch with IoMT application.

Figure 1 presents an overview of the IoMT-connected platform for smart healthcare monitoring. The IoMT system consists of four main modules with different functions: (1) A flexible biosensor patch with soft mechanical properties and high stretchability for long-term physiological signal recording. (2) Sensor data such as ECG, body temperature, GPS, body movement, and BP are collected and transmitted to a mobile gateway by a Bluetooth Low Energy (BLE) module. (3) The gateway functions as a bridge to connect vital paths with the health cloud data server for healthcare data storage and analytics. Finally, a mobile application and user interface (UI) website was developed for health data visualization and medical interaction. The proposed IoMT-connected system is an integration of unobtrusive biosensors and an H-IoT platform that contributes to preventive and occupational healthcare development.

### 2.2. Device Structure and Mechanical Performance

**Figure 2:**
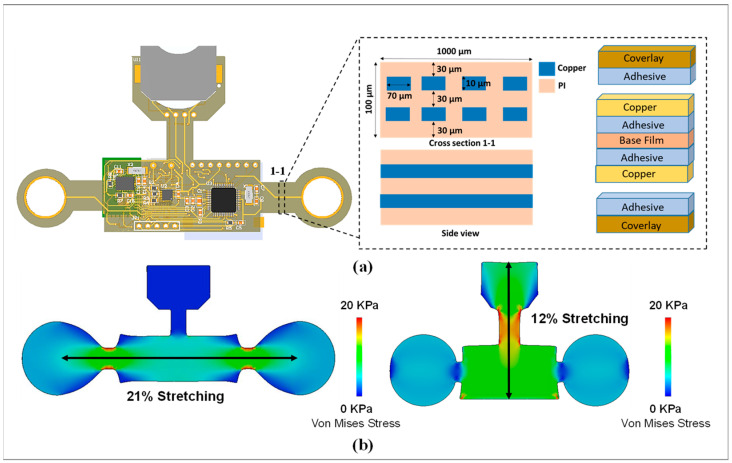
Design and mechanical properties of flexible biosensor patch. (**a**) Design and structure of the biosensor device. (**b**) Simulation result showing the stress between the vital patch and skin during bending deformations.

To maintain wearability and convenience for continuous and long-term physiological monitoring, a flexible biosensor patch must be fabricated with a compact size (<0.1 mm) and light weight (<5.0 g). This ensures maximum comfort and stability for long-term use on the curved skin surface of the chest. The overall layout of the vital patch was designed using an FPCB with double layers and mounted components (Figure 2a). The structure of a single-layer FPCB includes a thin film with four main classes of materials (i.e., base material, electrical conductors, cover lay, and adhesive material). For monitoring while attached to the skin, the mechanical properties and performance must be investigated to ensure the stretchability and compatibility of the flexible device on the curved surface of the skin. Furthermore, with high stretchability and soft mechanical properties, interfaces and interactions of the flexible device with the skin are minimal. Thus, skin irritation and discomfort due to long-term wearability can be prevented. For this study, SolidWorks (version 2020, SOLIDWORKS Corp., Waltham, MA, USA) was used to simulate and analyze the strain distribution of flexible biosensor patches. The mechanical performance of the device was quantified by the deformation capacity, which is expressed as the stretch ratio when the device is subjected to significant deformation. In the first case, two concentrated forces were applied at each end in the longitudinal direction of the device. Similarly, two concentrated forces applied at each end in the transverse direction were considered for the second case. It has been proven that 20 kPa is the threshold for sensory perception [34,35]. Therefore, the magnitude of the applied forces was optimized so that the stress values were 20 kPa for all cases to evaluate the degree of comfort of the device. Simulated results are shown in Figure 2b. The extreme scenarios of the stretch ratios are 21% and 12% for the first and second cases, respectively, indicating that the device could cause irritation and discomfort at the interface only if the device is subjected to very large deformations that are a departure from the normal operating status of the device. In conclusion, the flexible design of the biosensor patch ensures a high level of compatibility and stability with the skin surface for long-term attachment.

### 2.3. Hardware Architecture

**Figure 3:**
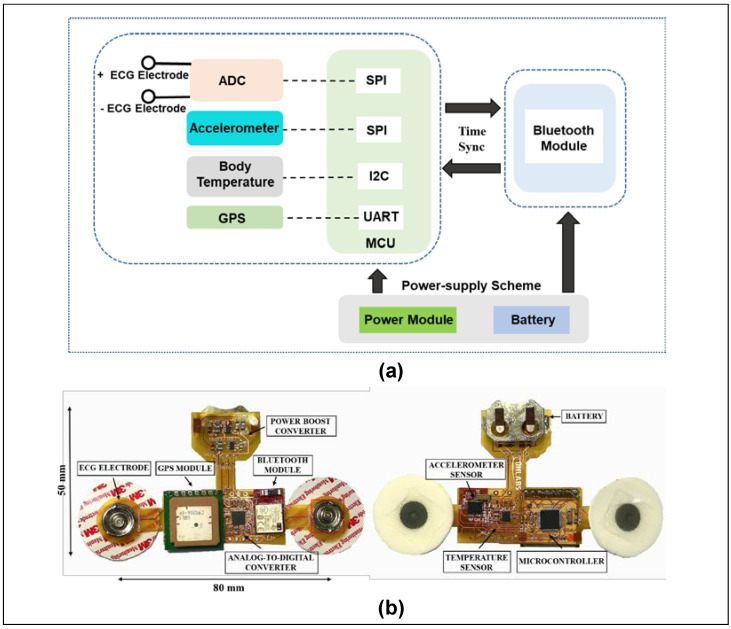
Hardware design of flexible biosensor patch for physiological monitoring. (**a**) Block diagram showing the operational scheme of the proposed device. (**b**) Prototype of the proposed biosensor.

Figure 3a highlights the operational scheme of the flexible and wireless biosensor patch. Following the block diagram, the flexible biosensor patch is divided into three main modules with different functions. The power supply module includes a rechargeable battery and power management integrated circuits (ICs) to regulate the required voltage (3.3 V or 2.2 V) for various components. The second unit is the biosensor module, including a PPG sensor, a 9-axis accelerometer, a clinical-grade temperature sensor, ECG, a GPS module, and a low-power microcontroller unit for health data acquisition and processing. The last component is a BLE module for wireless sensor data transmission. A prototype of the flexible biosensor patch is presented in Figure 3b. In the center module, a low-power microcontroller unit (PIC16LF19186, Microchip Technology Inc., Chandler, AZ, USA) and a multi-standard and BLE module (CC2650, Texas Instruments, Inc., Dallas, TX, USA) were selected as a control unit for processing and transmitting sensor data. In long-term health monitoring, optimization of power consumption is necessary to extend the operational time of the proposed device [31,32]. Thus, in this design, the input voltages of the microcontroller unit and BLE module were reduced to a minimal operating voltage range (2.2 V) and the low-power modes were configured for optimizing power consumption [36,37]. The biosensor module includes a wide-bandwidth 9-axis accelerometer (BNO055, Bosch Inc, Gerlingen, Germany), a human body temperature sensor (MAX30205, Maxim Integrated, San Jose, CA, USA), an ECG sensor that consists of two Ag/AgCl electrodes coated with hydrogel, and a GPS antenna module (PAM-7Q, u-Blox, Reston, VA, USA). The smart sensor BNO055 is a system-on-a-chip that integrates a triaxial 14-bit accelerometer, 16-bit gyroscope, and a triaxial geomagnetic sensor. With the advantages of rapid response time, low noise, and small size, the accelerometer sensor BNO055 is a suitable choice for human activity recording that requires long-term use and a minimized package size [38]. For ECG signal collection, two Ag/AgCl electrodes coated with hydrogel are affixed directly onto the chest of the subject. The 24-bit analog front-end for biopotential measurement (ADS1293, Texas Instruments, Inc. Dallas, TX, USA) was selected for analog-to-digital conversion [39]. The PAM-7Q GPS module, with its advantages of an embedded antenna, low power consumption, and simple interface, is an ideal choice for tracking user position [40]. Moreover, the MAX30205 temperature sensor, with high accuracy (±0.1 °C accuracy from 37 °C to 39 °C) and low-voltage operation (2.7 V to 3.3 V) is an ideal choice for body temperature measurement [41]. The power module includes a rechargeable lithium battery (ML2032, Maxell, Japan), and synchronous boost converters (TPS61322, TPS613221A Texas Instruments, Inc. Dallas, TX, USA) are used for power supply. The flexible biosensor patch is designed with a small size and low power consumption for long-term health monitoring.

### 2.4. IoT-Connected Healthcare Platform

**Figure 4:**
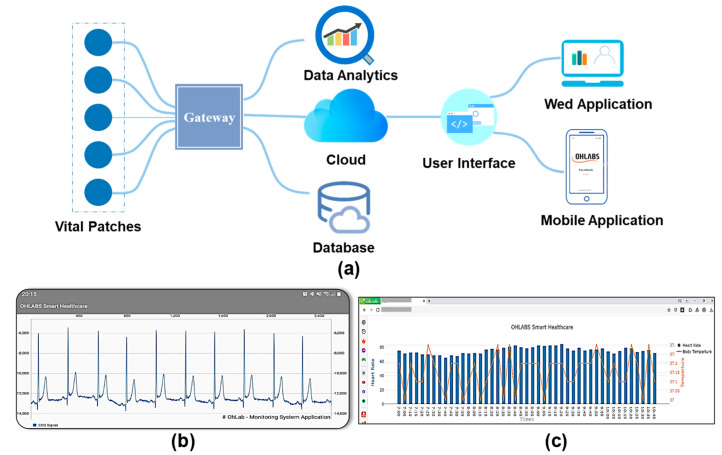
Implementation of the proposed wireless biosensors patch with IoMT-connected system. (**a**) Diagram of IoMT-connected platform. (**b**) Smartphone application. (**c**) Website application for remote healthcare management.

The advancement of smart healthcare IoMT applications has led to a paradigm shift in medicine 4.0 [42,43]. Herein, an IoT-connected healthcare platform including mobile and website (Web) user interfaces, a database server, an IoT gateway, and data flow is proposed for prognosis–health management. The vital signs data recorded by the biosensor patch are wirelessly transmitted to the mobile gateway using Bluetooth (BT) communication. A smartphone application was developed to visualize personal health data, including the user’s profile, physiological signals, and health status. A bio-connection between the biosensor patch and the smartphone app was established for the device parameter configuration and display (battery status, sample rate, etc.). Health data are automatically sent to the server for data storage and health analysis. The mobile gateway is responsible for the bio-connection between the IoMT cloud database and the biosensor patch. The health data are transferred from the gateway to the server using a message queuing telemetry transport protocol [44]. The Firebase cloud server was selected in the IoMT-connected platform for real-time data storage and management [45]. A large amount of vital sign data through the sensor patches were collected for data analytics. The health data such as biometric information, physiological parameters were analyzed for better diagnosis and health deterioration risks identification. The integration of medical data analytics and IoMT play important roles in tackling the issue of increasing health information extraction and exchange. The health data will be visualized on a smartphone application, or a web based on a local UI for remote healthcare management (Figure 4b,c). Medical professionals and healthcare staff can access the web application for remote patient health status monitoring and emergency rescue. The IoMT-connected healthcare platform with multiple biosensor patch communication will lead to the development of a cost-effective and smart healthcare system in the near future [46].

## 3. Results

### 3.1. Health Status Monitoring

**Figure 5:**
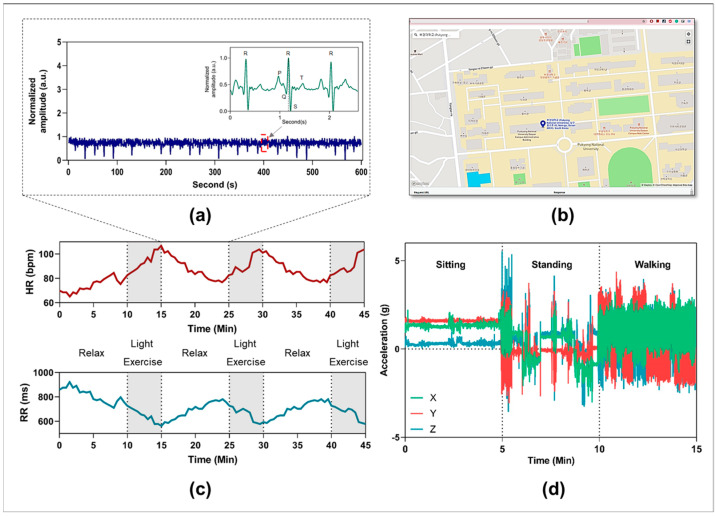
Representative data were collected by the proposed vital patch device. (**a**) Representative ECG waveforms were collected from one subject in 10 min. (**b**) Representative GPS position of one subject based on using the proposed monitoring device. (**c**) Monitoring heart rate and RR value of one subject with different light exercise sessions. (**d**) *x*-, *y*-, and *z*-axis accelerometry signals recording from one subject with different daily activities.

The performance of the flexible biosensor patch for monitoring cardiovascular parameters and physiological responses was evaluated. Among the physiological responses of the body, the ECG signal is one of the most important vital signals [47]. ECG waveforms can dynamically and directly reflect cardiac conditions and cardiovascular disease (CVD) [48]. The simultaneous monitoring of ECG signals and cardiac cycle reference (QRS complex) is essential for early diagnosis and prompt intervention in CVDs [49]. Here, experiments were designed to evaluate the ability of the flexible biosensor patch in real time, continuously collecting ECG signals and cardiovascular responses with physical activity. The volunteers were required to perform three light cycling exercise sessions for 45 min with a 10-min rest period between each session. An ECG waveform with QRS analysis recorded in the relaxed phase is shown in Figure 5a. Figure 5c illustrates the physiological responses, including changes in heart rate and RR interval during different light exercise sessions. Furthermore, body position and accelerometry signals can be continuously monitored using a high-bandwidth, 9-axis accelerometer. The *x*-, *y*-, and *z*-axis accelerometry data recorded from one subject during different daily activities are shown in Figure 5d. Based on the results, the acceleration (g) in the *x*, *y*, and *z*-axis showed considerable differences in the cases of sitting, standing, and walking, which is important for tracking body movement and fall detection in daily activities [50]. Moreover, a GPS module is included in the device to track user position and enable emergency rescue. In the case of abnormal situations, a short message service (SMS) alert including the user’s profile and GPS data will be automatically sent to relevant medical professionals or family members [51]. Figure 5b shows real-time GPS locations of one subject using the proposed wireless vital patch. In summary, the feasibility of such biosensor patches in simultaneously and continuously recording multiple physiological parameters and biomarkers in real-life scenarios was demonstrated. Monitoring the body’s response in daily activities can be beneficial for the early detection and prevention of abnormal cardiovascular changes [50,51].

### 3.2. Advanced Use Cases in BP Estimation

**Figure 6:**
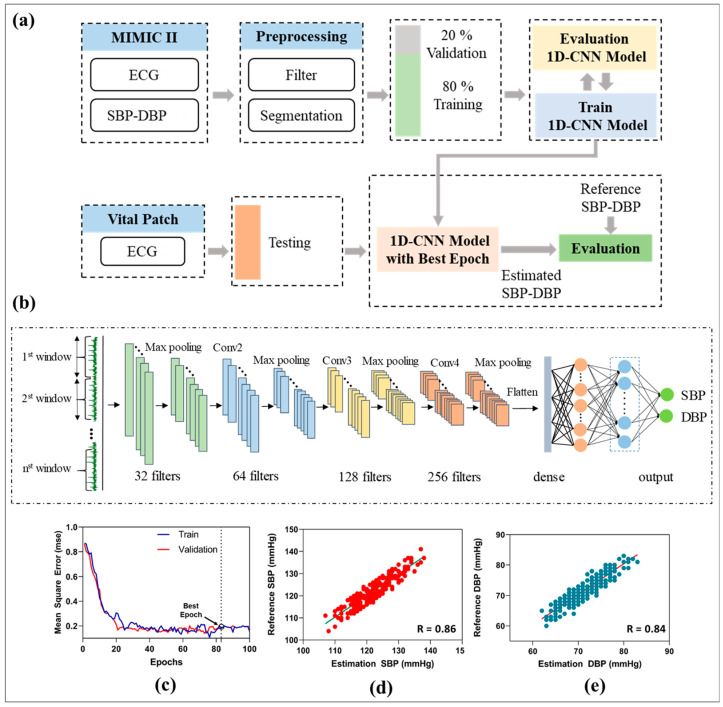
Overview of proposed BP estimation method using single-channel ECG. (**a**) Overview of the proposed BP estimation method. (**b**) The architecture and structure of 1D-CNN. (**c**) Performance of the proposed neural network with mean squared error. (**d**) Correlation plots of estimated SBP with reference SBP. (**e**) Correlation plots of estimated DBP with reference DBP.

Continuous BP measurement is necessary to prevent hypertension and cardiovascular diseases [16,17]. This study proposes a deep learning model using 1D-CNN and single-channel ECG signals for a continuous BP estimation. An overview of the developed method for BP estimation is shown in Figure 6. A 1D-CNN and two fully connected layers are combined to extracted morphological and rhythmic features from ECG signals. The outputs from the fully connected layers are the predicted results of systolic and diastolic blood pressures. In this study, ECG and arterial BP (ABP) signals were extracted from Physionet’s Multiparameter Intelligent Monitoring in Intensive Care (MIMIC) II dataset [52]. The ECG and ABP signals were acquired at a sampling rate of 125 Hz and divided into two rows for further processing. Notably, the raw ECG signals are contaminated with different artifacts, noise power frequency interference, baseline drift, and contract interference. Therefore, a low-pass filter with a cut-off frequency of 50 Hz and wavelet transformation were applied to process and remove the high-frequency interference and baseline drift of the raw ECG signals [39]. Next, a sliding window with no overlap was used to segment the ECG and ABP signals into a fixed length of 128 points. Peaks and inverse peaks were extracted from the simultaneous ABP waveform, SBP, and DBP calculations. The average values of SBP and DBP in each data frame were calculated and used as inputs for the training process. After the sampling step, 80% of the data were divided for training the model, and 20% were selected for model performance evaluation. Next, the ECG signals and corresponding BPs were fitted into the training 1D-CNN for model training and evaluation [53]. The proposed network architecture of the BP estimation model (Figure 6b) consists of four convolutional layers, four max-pooling layers, and two fully connected layers that output the estimated SBP and DBP. The number of filters was set to 32, 64, 128, and 256. Finally, 128 features are learned through the proposed 1D-CNN model servers as input in this step and connected to two fully connected layers. The numbers of nodes in each layer of the fully connected layers were 32 and 16, respectively. A rectified linear unit was selected as the activation function. Finally, a linear function was used as the final feature for SBP and DBP estimation. The mean squared error was chosen as the error function to evaluate the accuracy of the developed deep learning model. The proposed model was trained with a batch size of 32, learning rate of 0.01, and adaptive moment estimation (Adam). This study uses mean square error (MSE) as a loss function in the training process. The model was optimized at epoch 83 (best epoch), with a training and validation mean squared error of 0.18 (Figure 6c).

To assess the performance of the proposed deep learning architecture for continuous BP estimation using a flexible biosensor device, five young healthy volunteers (27 ± 3 years old, 70 ± 5 kg) with no history of CVDs and hypertension were recruited to collect ECG signals and reference BP measurements. The subjects were required to sit and rest to collect the bio-signals. ECG waveforms were collected from the developed vital patch, while reference SBP and DBP were recorded using commercial ambulatory BP monitoring (Oscar 2, SunTech, Carlsbad, CA, USA). The correlation between the reference BP and the estimated BP using the proposed neural network model is shown in Figure 6c,d. The results showed a high correlation coefficient (r = 0.86 for SBP, 0.84 for DBP), which fulfills the requirements of the Association for the Advancement of Medical Instrumentation (AAMI) and the British Hypertension Society (BHS) standards for BP monitor certification [54,55]. Alternatively, the proposed BP estimation model combined with the flexible biosensor patch exhibited good performance with an acceptable requirement for non-invasive BP estimation.

## 4. Discussion

In this study, we demonstrated the feasibility of a flexible, skin-interfacing, and wireless biosensor patch with an IoMT-connected healthcare platform for monitoring vital signs and body responses. In fact, the flexible biosensor patch was designed with high levels of mechanical stretchability and reliability in the case of a curved skin surface to enhance wearability for long-term cardiac monitoring outside of clinical environments [30,34]. Another advantage of flexible devices is their ability to capture body orientation and movement information, which are relevant in terms of fall detection [50]. Moreover, the GPS location of the user is recorded when using the wireless device, which is valuable for tracking user location, and to facilitate emergency rescue in cases of abnormal situations [43,51]. The successful performance of the biosensor patch in monitoring physiological data has the potential to provide insight into cardiac conditions and CVD diagnosis [1,2,3].

With the advantages of data accessibility, data sensing, and communication, IoT is becoming a key feature for the development of smart cities, manufacturing industries, consumer services, etc. The integration of IoT in healthcare applications is referred to as H-IoT. H-IoT is a subset of IoT systems that are increasingly employed for diagnosis–treatment methodology and the prognosis–health management approach [42]. In general, both IoT and H-IoT refer to a network that is embedded with smart sensors, embedded devices, and processing ability for data communication and integrity. However, there are several differences between the generic IoT and H-IoT [13,43]. Firstly, the underlying technology for IoT is a wireless sensor network (WSN), while that for H-IoT is identified as a body sensor network (BSN). A BSN is a network of wearable or implantable biosensors placed on the human body for healthcare applications [13]. Secondly, the current widespread applications of IoT are in maintenance management, smart grid, and saving, as well as traffic monitoring, whereas H-IoT only focuses on medical applications, including fitness tracking and cardiovascular diseases [14]. Another difference between generic IoT and H-IoT is the energy and power consumption, in which the former deploys in a large geographical area and utilizes a large amount of energy, while the latter deploys in a small geographical area and uses the energy harvested from human self-generated heat, motion, and battery [56]. Moreover, the nodes of generic IoT are varied in size and stability, while those of H-IoT are miniature and mobile [34]. The generic IoT allows sensor deployment and maintains data integrity, whereas deploying sensors in H-IoT is more difficult and requires data preservation and transmittance at a high integrity level [43]. For the IoMT-connected platform, a BLE module is employed for wireless connectivity between the biosensor device and a mobile gateway, which transmits health data to an IoT cloud server for data storage and management. Real-time physiological measurements are visualized on a smartphone app or a UI website for real-time monitoring and access to patient health data. A mobile gateway functions as an intelligent central link between the biosensor patch and the IoT cloud server for data transmission and synchronization to enhance an efficient end-to-end interaction between patients, family members, and medical staff in real time. The development of the IoMT-connected platform will facilitate a loosely coupled connectivity between the user and their caregivers at different locations, thus reducing healthcare costs, length of hospital stay, and improving medical outcomes [42,43,46]. This will also contribute to healthcare and lifestyle behavioral changes in the near future [46].

In this research, we proposed a cuffless continuous BP measurement model based on a deep CNN model employing single-channel ECG signals. A BP estimation algorithm based on pulse arrival time, pulse wave velocity, and pulse transit time for non-invasive BP measurement has been proposed in numerous studies, but most multiparameter-based methods require a complex setup of ECG and PPG signals [15,16,17,18]. Yan et al. developed a BP estimation method using a single-channel PPG signal and support vector machine as a regression model involving the estimated and reference BP [57]. However, the BP estimation model based on a single source of PPG signals exhibits inferior performance owing to the effects of noise, motion artifacts, and sensor placement [30,31]. Motivated by this, an ECG–BP estimation algorithm was proposed. The close association between ECG signals and morphological changes to BP has been discussed previously in a small number of studies [32,33]. Compared to PPG signals, ECG signals are easier to acquire with high-quality, reliability, and low-noise effects [33]. The emergence of artificial intelligence in solving medical problems and signal processing has led to the development of many BP estimation models with different traditional machine learning models, such as an ensemble of trees, random forests, regression trees, and support vector machine (SVM) [15,16,17]. Deep neural networks, with advantages in complex non-linear relationships, have proven to be a powerful method for improving the accuracy of BP estimation models [20,21,22,23]. The combined deep learning architecture-based multitasking model shows a better performance in comparison with the BP estimation model using a single model due to the sequence relationship between pulse pressure and PAT [58]. Certain limitations should be addressed in future developments. First, the experimental subjects of the study should be expanded to include overweight, elderly, and hypertensive subjects. Second, the effects of factors such as CVD, drug state, and caffeine levels should be considered in future studies. Third, more advanced data pre-processing procedures could be leveraged to remove noise effects and improve the stability of BP models. Lastly, the future development can incorporate the biosensors patch with wireless power transfer technology and a flexible printed coil array for a comprehensive skin-worn sensing device [6,7].

## 5. Conclusions

In short, a flexible, wireless, and wearable biosensor patch with skin-conformal attachment and multiple biosensor integration for monitoring different vital signs is introduced in this study. The proposed flexible biosensor device consists of sensor, center, power, and biosensor modules, which are connected to a flexible printed circuit board (FPCB) for wearable health monitoring of body temperature, body movement, heart rate, and BP. The flexible biosensors patch is designed with a compact size (<0.1 mm) and to be lightweight (<5.0 g) to maintain wearability and convenience for long-term use. The simulation results show an acceptable stretch ratio (21%, and 12%) between the flexible device and skin during bending deformation. The stress value was optimized below the threshold for sensory perception (20 kPa) to increase the degree of comfort and the stability of the device with the skin surface for long-term attachment. In addition, an IoMT platform with a cloud server, smartphone application, and remote monitoring website is demonstrated for prognosis–health management. The applicability of the flexible biosensor patch for monitoring cardiovascular parameters and physiological responses was demonstrated. The ECG waveform and cardiovascular responses in three light cycling exercise sessions for 45 min with a 10-min rest period between each session were recorded. Moreover, the accelerometry signals, body movement, and user position were simultaneously and continuously recorded for smart healthcare applications. In addition, by leveraging the advantages of a deep neural network for a regression problem, a BP estimation model based on a one-dimensional convolutional neural network (1D-CNN) and single-channel ECG signals with superior performance is proposed. The proposed BP estimation model presented a high correlation coefficient for BP prediction (r = 0.86 for SBP, 0.84 for DBP), which satisfies the requirements of the AAMI and BHS for BP monitor devices. In summary, this study highlighted the performance of a flexible and wireless biosensor patch with an IoT-connected healthcare platform for remote healthcare monitoring external to the clinical environment.

## Data Availability

The data presented in this study are available on request from the corresponding author.
